# GABA_B_ receptor ligands for the treatment of alcohol use disorder: preclinical and clinical evidence

**DOI:** 10.3389/fnins.2014.00140

**Published:** 2014-06-06

**Authors:** Roberta Agabio, Giancarlo Colombo

**Affiliations:** ^1^Department of Biomedical Sciences, University of CagliariMonserrato, Italy; ^2^Section of Cagliari, Neuroscience Institute, National Research Council of ItalyMonserrato, Italy

**Keywords:** GABA_B_ receptor, baclofen, positive allosteric modulators, alcohol use disorder, animal models of alcohol use disorder

## Abstract

The present paper summarizes the preclinical and clinical studies conducted to define the “*anti*-alcohol” pharmacological profile of the prototypic GABA_B_ receptor agonist, baclofen, and its therapeutic potential for treatment of alcohol use disorder (AUD). Numerous studies have reported baclofen-induced suppression of alcohol drinking (including relapse- and binge-like drinking) and alcohol reinforcing, motivational, stimulating, and rewarding properties in rodents and monkeys. The majority of clinical surveys conducted to date—including case reports, retrospective chart reviews, and randomized placebo-controlled studies—suggest the ability of baclofen to suppress alcohol consumption, craving for alcohol, and alcohol withdrawal symptomatology in alcohol-dependent patients. The recent identification of a positive allosteric modulatory binding site, together with the synthesis of *in vivo* effective ligands, represents a novel, and likely more favorable, option for pharmacological manipulations of the GABA_B_ receptor. Accordingly, data collected to date suggest that positive allosteric modulators of the GABA_B_ receptor reproduce several “*anti*-alcohol” effects of baclofen and display a higher therapeutic index (with larger separation—in terms of doses—between “*anti*-alcohol” effects and sedation).

## Introduction

Alcohol abuse and dependence are severe mental disorders characterized by bouts of compulsive and uncontrolled alcohol consumption, and an inability to cut down drinking despite the knowledge of its negative consequences (American Psychiatric Association, 2000). These frequent disorders are related to chronic medical conditions, car crashes, domestic violence, fetal alcohol syndrome, neuropsychological impairment, economic costs, loss of productivity, and psychiatric comorbidity (see Rehm et al., 2009; Nutt et al., 2010). Up to the penultimate edition of the Diagnostic and Statistical Manual of Mental Disorders (DSM-IV-TR) (American Psychiatric Association, 2000), alcohol abuse and dependence were classified as independent alcohol use disorders (AUDs); in the last DSM edition (DSM-5), they have been joined into a single mental disorder, alcohol use disorder, or AUD (American Psychiatric Association, 2013). Diagnostic criteria for AUD include those listed for the diagnosis of dependence and abuse, together with a new criterion, a subjective experience of wanting to consume alcohol, also defined “craving” (Tiffany and Wray, 2012).

The aims of AUD treatment consist in achieving abstinence, or reducing alcohol consumption, reducing the frequency and severity of relapses, and improving psychological and social functioning. The process involved in acquiring and maintaining abstinence from alcohol may require a first phase known as “detoxification” aimed at decreasing withdrawal symptoms (when present), and a second “rehabilitation” phase, aimed at maintaining the motivation to abstain, developing an alcohol-free lifestyle, and reducing the risk of relapse. Treatment to achieve these aims includes psychological and pharmacological therapies. Psychological and social interventions (including *Alcoholics Anonymous* and various counseling approaches) are usually considered core treatment options (see Schuckit, 2009). However, these approaches have demonstrated only a moderate success rate. Pharmacotherapy should be used to increase the efficacy of non-pharmacological approaches. Unfortunately, the few available AUD medications display only moderate efficacy, with several limitations to their clinical use, including poor compliance and even abuse liability (see Chick and Nutt, 2012; see Franck and Jayaram-Lindström, 2013).

Over the last 15 years, multiple lines of experimental and clinical evidence have suggested a role for the GABA_B_ receptor in the control of several alcohol-related behaviors (for reviews on the GABA_B_ receptor, see Bettler et al., 2004; Couve et al., 2004; Emson, 2007). Pharmacological activation of the orthosteric binding site of the GABA_B_ receptor has repeatedly been reported to suppress alcohol drinking and alcohol withdrawal syndrome (AWS) in rodents and human alcoholics, as well as alcohol reinforcing and motivational properties in rodents and non-human primates and craving for alcohol in human alcoholics. These data have led to the development of the prototypic GABA_B_ receptor agonist, baclofen (used for more than 50 years in the treatment of spasticity), as a promising, novel pharmacotherapy for AUD.

More recently, identification of a binding site of positive allosteric modulation (topographically distinct from the orthosteric binding site) has provided a new strategy for pharmacological manipulation of the GABA_B_ neurotransmission. Activation of this binding site—by a class of agents known as positive allosteric modulators of the GABA_B_ receptor (GABA_B_ PAMs)—augments the affinity of the GABA_B_ receptor for GABA and agonists and synergistically potentiates their effects (see Froestl, 2010). GABA_B_ PAMs are devoid of substantial intrinsic agonistic activity in the absence of GABA; they do not perturb receptor signaling on their own, but potentiate the effect of GABA only in those synapses where and when endogenous GABA has been released (see Froestl, 2010). Accordingly, GABA_B_ PAMs are expected to produce fewer side effects and lower tolerance. Rodent data confirm that GABA_B_ PAMs reproduced several *in vivo* effects of baclofen, displaying a notably larger separation between the “desired” pharmacological effects (e.g.: anxiolysis, “*anti*-addictive” effects) and “unwanted,” or adverse, effects (e.g., sedation, motor-incoordination). For these reasons, GABA_B_ PAMs currently represent a major step forward in the pharmacology of the GABA_B_ receptor.

The present paper is aimed at reviewing the lines of preclinical and clinical evidence featuring baclofen and GABA_B_ PAMs as potentially effective pharmacotherapies for AUD.

## Preclinical data

### Baclofen

The vast majority of data presently available have been collected with baclofen; some of these results have subsequently been reproduced with additional GABA_B_ receptor agonists, including CGP44532 and SKF97541 (also known as CGP35024).

#### Acquisition of alcohol drinking

A first set of studies investigated the effect of acute or repeated administration of baclofen on alcohol intake in rats or mice exposed to the conventional, homecage 2-bottle “alcohol vs. water” choice regimen. Under this experimental procedure, animals are exposed to the choice between a relatively low concentrated alcohol solution (usually 10%, v/v) and water and freely allowed to consume alcohol. This procedure —largely used because of its relative simplicity—provides information on the mere consumption of alcohol. It possesses however considerable predictive validity, especially when applied to rats selectively bred for high alcohol preference and consumption.

When repeatedly (once daily for 10 consecutive days) administered over the initial period of exposure to alcohol, baclofen (1–3 mg/kg, i.p.) has been reported to completely block the acquisition of alcohol drinking behavior in selectively bred, Sardinian alcohol-preferring (sP) rats (Colombo et al., 2002). Acquisition of alcohol drinking occurred only once treatment with baclofen had been interrupted. These data suggest that activation of the GABA_B_ receptor effectively blocked discovery and experience of those psychopharmacological effects of alcohol that sustain alcohol drinking behavior in sP rats. Reduction in alcohol intake was associated to a compensatory increase in daily water intake, so that total daily fluid intake remained unchanged; these data suggest the selectivity of baclofen effect on alcohol intake and lead to reasonably exclude that the suppressing effect of baclofen on alcohol intake was secondary to possible motor-incapacitating or sedative effects (that would disrupt the normal rates of drinking).

These data have subsequently been replicated with CGP44532: its repeated administration (0.03–1 mg/kg, i.p., once daily for 10 consecutive days) completely blocked acquisition of alcohol drinking in sP rats (Colombo et al., 2002).

#### Maintenance of alcohol drinking

More numerous studies have investigated the effect of baclofen on alcohol intake in alcohol-experienced rats (i.e., rats in which the consumption of pharmacologically relevant doses of alcohol was already well consolidated before the start of treatment with baclofen; these alcohol-experienced rats are thought to model the “maintenance” or “active drinking” phase of human alcohol dependence). Acute or repeated (once daily for 4–14 consecutive days) administration of doses of baclofen ranging between 1 and 10 mg/kg (i.p.) suppressed, in a dose-related fashion, alcohol intake in selectively bred sP (Colombo et al., 2000) and University of Chile bibulous (UChB) (Quintanilla et al., 2008) rats, unselected Long Evans (Daoust et al., 1987; see however Smith et al., 1999) and Wistar (Stromberg, 2004) rats, and C57BL/6N (Peters et al., 2013) and Swiss (Villas Boas et al., 2012) mice. These results suggest the ability of baclofen to decrease alcohol intake in rats and mice displaying consolidated and relatively high levels of alcohol consumption.

#### Alcohol deprivation effect

Additional studies used the free-choice regimen to investigate the effect of treatment with baclofen in a rodent model of alcohol relapse named “alcohol deprivation effect” (ADE). ADE is defined as the temporary increase in voluntary alcohol intake—often doubling the regular intake—occurring after a period of forced abstinence, or deprivation, from alcohol (see Martin-Fardon and Weiss, 2013). An initial study (Colombo et al., 2003a) employed the 2-bottle “alcohol (10%, v/v) *vs* water” choice regimen and found that acute administration of baclofen (1-3 mg/kg, i.p.) to sP rats previously deprived of alcohol for 7 consecutive days resulted in the complete suppression of the extra amount of alcohol consumed during the first hour of re-access (the time period during which ADE is maximal in sP rats). In a subsequent study (Colombo et al., 2006), sP rats were exposed to a 4-bottle “alcohol vs. water” choice regimen with multiple alcohol concentrations (10, 20, and 30%, v/v). Acute injection of baclofen (1 mg/kg, i.p.) at the end of the 14-day alcohol-deprivation period resulted in the complete suppression of both aspects of the augmented demand for alcohol: (a) increase in the amount of alcohol consumed; (b) shift in preference for the highest concentrated alcohol solution (that would result in a more rapid absorption of alcohol and faster perception of its psychopharmacological effects). Notably, in both studies water and food intake, as well as spontaneous locomotor activity, were not altered by baclofen treatment, suggesting that its suppressing effect was selective for alcohol and occurred at doses devoid of any sedative effect. Similar data have been collected with CGP44532: its acute administration (0.03–1 mg/kg, i.p.) selectively blocked indeed ADE in sP rats re-exposed to alcohol after a 14-day period of alcohol deprivation (Carai et al., 2005). These data suggest a role for the GABA_B_ receptor in the neural substrate controlling relapse-like drinking in rats.

#### Binge-like alcohol drinking

Baclofen has also been found to suppress alcohol intake in two mouse models of binge drinking. The first study (Moore and Boehm, 2009) used the experimental model *Drinking in the Dark* (DID), based on the exposure of alcohol-consuming C57BL/6J mice to daily drinking sessions of 2 h (3 h into the dark phase of the light-dark cycle) with a single bottle containing 20% (v/v) alcohol. Acute microinjection of baclofen (0.01 and 0.02 μg) into the anterior, but not posterior, portion of the ventral tegmental area (VTA) resulted in a marked reduction of alcohol intake. Baclofen microinjection did not alter the intake of water or sweetened water, demonstrating the drug selectivity on alcohol intake. The second study (Tanchuck et al., 2011) used the experimental model *Scheduled High Alcohol Consumption*, in which Withdrawal Seizure Control mice were exposed to 30-min drinking sessions every third day with 5% (v/v) alcohol. Again, acute administration of baclofen (2.5–5 mg/kg, i.p.) markedly reduced alcohol intake.

#### Operant self-administration of alcohol

Several studies have also tested the capacity of baclofen to suppress operant, oral alcohol self-administration in rats. Under these procedures, alcohol is made available to rats *via* completion of a behavioral response (usually, responding on a lever for a given number of times), to allow the reinforcing and motivational properties of alcohol to be assessed (in addition to its mere consumption). Operant procedures differ from the alcohol-drinking procedures in that alcohol is not “freely” available and a specific amount of “work” is required to access alcohol. Baclofen has been tested under the three most commonly used procedures of alcohol self-administration: (a) fixed ratio (FR) schedule of reinforcement, in which the response requirement (RR; i.e., the “cost” of each alcohol presentation in terms of number of responses on the lever) is predetermined and kept fixed throughout the session (providing a measure of both alcohol consumption and reinforcing properties of alcohol); (b) within-session progressive ratio (PR) schedule of reinforcement, in which—over the same single session—RR is progressively increased after the delivery of each reinforcer, and the lowest ratio not completed (named breakpoint) is taken as measure of motivational properties of alcohol; (c) within-session extinction responding (ER), in which lever-responding is never reinforced: the highest number of lever-responses (named ER) is taken as measure of motivational properties of alcohol.

The studies using the FR (varying from FR1 to FR4) schedule of reinforcement have reported that acute (often using a Latin square design) and repeated (3 consecutive daily self-administration sessions) treatment with baclofen (0.5–5.6 mg/kg, i.p., in rats; 1–17 mg/kg, i.p., in mice) reduced both number of lever-responses for alcohol and amount of self-administered alcohol in (a) selectively bred alcohol-preferring sP (Maccioni et al., 2005, 2012), Indiana P (Liang et al., 2006; Maccioni et al., 2012), and Alko Alcohol (AA) (Maccioni et al., 2012) rats, (b) unselected Long-Evans (Anstrom et al., 2003; Janak and Gill, 2003) and Wistar (Petry, 1997; Walker and Koob, 2007; Dean et al., 2011) rats, and (c) C57BL/6J mice (Besheer et al., 2004). In close agreement with this large set of data on baclofen, acute treatment with SKF97541 (0.1–1 mg/kg, i.p.) reduced operant alcohol self-administration in C57BL/6J mice (Besheer et al., 2004).

Similar data were also collected in the PR and ER experiments: treatment with baclofen (1–3 mg/kg; i.p; administered using a Latin square design) (a) reduced the breakpoint for alcohol in sP (Maccioni et al., 2008b, 2012), P (Maccioni et al., 2012), AA (Maccioni et al., 2012) and Wistar (Walker and Koob, 2007) rats as well as (b) virtually completely suppressed ER in sP rats (Colombo et al., 2003b).

Taken together, data from alcohol self-administration studies suggest that treatment with non-sedative doses of baclofen effectively reduced—beside alcohol consumption—the reinforcing and motivational properties of alcohol. Since operant procedures of alcohol self-administration in rodents possess predictive validity for human craving for alcohol (see Markou et al., 1993), these data bear translational relevance. In terms of translation “from lab bench to bedside,” it may be worth noting that baclofen resulted to be more potent and effective in those rats (either selectively bred or made physically dependent on alcohol) seeking and taking larger amounts of alcohol (Walker and Koob, 2007; Maccioni et al., 2012). This view shares some similarities with clinical data. Indeed, as stated below, the hypothesis that baclofen may be more effective in patients affected by severe forms of AUD has been advanced to reconcile the discrepancy observed among some clinical surveys: surveys recruiting patients affected by severe alcohol dependence provided stronger evidence on the suppressing effect of baclofen on alcohol craving and consumption than those studies including individuals affected by moderate AUD.

When investigated, the selectivity of the reducing effect of baclofen on alcohol self-administration was relatively modest, as treatment with baclofen tended to also reduce self-administration of and ER for sucrose or saccharin solutions or regular food pellets (used as alternative, non-drug reinforcers) with magnitude comparable to that observed in the “alcohol” experiments (Anstrom et al., 2003; Colombo et al., 2003b; Janak and Gill, 2003; Maccioni et al., 2005, 2008b, 2012).

#### Reinstatement of alcohol-seeking behavior

Operant procedures of alcohol self-administration can be profitably used also for studies of reinstatement of alcohol-seeking behavior. In this procedure, previously extinguished, unreinforced lever-responding for alcohol is resumed—or reinstated—by alcohol-related stimuli, alcohol itself, stressors, or drugs such as nicotine and cannabinoids. Reinstatement of alcohol-seeking behavior represents an experimental model of loss of control over alcohol and relapse episodes occurring in alcohol-dependent patients (see Martin-Fardon and Weiss, 2013). In agreement with the results of the ADE experiments (see above), acute treatment with baclofen (3 mg/kg, i.p.) markedly reduced lever-responding in sP rats initially trained to lever-respond for alcohol under an FR4 schedule of reinforcement, then subjected to an ER phase, and finally to a single reinstatement session in which lever-responding was effectively reinstated by presentation of an alcohol-associated stimulus complex (Maccioni et al., 2008a).

A recent study (Duke et al., 2014) has generated the first set of data on the “*anti*-alcohol” effects of baclofen in non-human primates, providing a powerful translational bridge between rodent and human studies. Adult, male baboons were trained under a chained schedule of reinforcement, made by three linked components: sequential completion of the RR of each component ultimately resulted in availability of oral alcohol (4%, w/v). Acute, intramuscular injection of baclofen (1.8–2.4 mg/kg) produced significant decrements in lever-responding for alcohol, number of alcohol drinks, and amount of self-administered alcohol. An additional experiment found that baclofen also decreased lever-responding in a single ER session. As in most rodent studies, selectivity was relatively modest, as baclofen reduced the self-administration of an alternative, non-drug reinforcer (orange-flavored beverage) with limited dose separation in comparison to the “alcohol” experiments. Nevertheless, these results extend to non-human primates the capacity of baclofen to attenuate the reinforcing effects of alcohol.

#### Alcohol stimulating and rewarding effects

Beside the several, above-mentioned studies on baclofen effect on alcohol-seeking and taking behaviors, additional rodent studies have investigated the effect of treatment with baclofen on other alcohol-related responses and behaviors. Specifically, it has been reported that acute administration of 1–3 mg/kg baclofen (i.p.) suppressed the stimulation of locomotor activity induced in alcohol-preferring UChB (Quintanilla et al., 2008) and unselected Sprague-Dawley (Arias et al., 2009) rats and mice belonging to different strains/lines (including NMRI, BALB/c, DBA/2J, and selectively bred FAST) (Cott et al., 1976; Humeniuk et al., 1993; Shen et al., 1998; Broadbent and Harless, 1999; Chester and Cunningham, 1999; Boehm et al., 2002; Holstein et al., 2009) by treatment with low-to-moderate doses of alcohol. These data are of relevance, as locomotor stimulation induced by a psychoactive drug, including alcohol, has been proposed to model its euphorigenic properties. Hyperlocomotion in rats and mice, and stimulation and euphoria in humans are indeed homologous phenomena, mediated by activation of common neural systems (see Wise and Bozarth, 1987). Baclofen capacity to suppress alcohol-stimulated locomotor activity in rodents may therefore be predictive of a suppressant effect on alcohol stimulating and euphorigenic actions in humans.

Acute microinjection of non-sedative doses of baclofen into the VTA suppressed alcohol-induced conditioned place preference in mice (Bechtholt and Cunningham, 2005); since conditioned place preference is a behavioral technique validated to investigate the rewarding properties of psychoactive drugs (see Tzschentke, 2007), these data suggest that pharmacological activation of the GABA_B_ receptor may suppress alcohol “reward.”

#### Hypotheses on the mechanism(s) of action

In terms of the mechanism(s) of action underlying the “*anti*-alcohol” effects of baclofen, data collected to date suggest the involvement of (a) GABA_B_ receptors located in the VTA both pre-synaptically (on the cell body of dopamine neurons) and post-synaptically (on the terminals of glutamatergic afferent neurons) (see Bowery et al., 1987) and (b) the mesolimbic “reward” dopamine system, whose neurons originate in the VTA, project into the nucleus accumbens (NAc), and mediate the reinforcing, motivational, stimulating, and rewarding properties of alcohol (see Weiss and Porrino, 2002). This hypothesis is based on several lines of experimental evidence demonstrating that acute microinjections of non-sedative doses of baclofen into the VTA suppressed: (a) alcohol binge-like drinking in mice (Moore and Boehm, 2009); (b) alcohol-seeking behavior in rats (Leite-Morris and Czachowski, 2006; Leite-Morris et al., 2008); (c) alcohol-induced stimulation of dopamine release in the rat NAc (K.A. Leite-Morris, personal communication); (d) alcohol-stimulated locomotor activity in mice (Boehm et al., 2002; Leite-Morris, 2004); (e) alcohol-induced place preference in mice (Bechtholt and Cunningham, 2005). Together, these results suggest that activation of the GABA_B_ receptors in the VTA results in inhibition of alcohol-induced firing of dopaminergic neurons, alcohol-stimulated dopamine release in the NAc, and—in turn—multiple dopamine-controlled, alcohol-related responses and behaviors.

#### Alcohol withdrawal syndrome

Several studies demonstrated that treatment with baclofen (1.25–20 mg/kg, i.p.) was also effective in suppressing multiple signs of AWS, including (a) increase in alcohol self-administration in Wistar rats made alcohol-dependent by prolonged exposure to alcohol vapors (Walker and Koob, 2007), suggesting the ability of baclofen to suppress the negative reinforcing properties of alcohol, (b) anxiety-related behaviors in Lister and Sprague-Dawley rats made alcohol-dependent by prolonged, forced exposure to an alcohol-containing diet (File et al., 1991; Knapp et al., 2007), and (c) tremors and seizures in Wistar rats made alcohol-dependent by the repeated administration of intoxicating amounts of alcohol (Colombo et al., 2000). As detailed below, these data depicting the capacity of baclofen to suppress multiple signs of AWS in rats have found a remarkable translational potential in several clinical studies. In terms of the mechanism(s) of action, activation of the GABA_B_ receptors likely counterbalances the enhanced function of glutamate excitatory neurotransmission associated to AWS (Colombo et al., 2000; Knapp et al., 2007).

### Positive allosteric modulators

#### Alcohol drinking

Virtually all studies conducted to date demonstrate that the “*anti*-alcohol” effects of baclofen and other GABA_B_ receptor agonists may be reproduced by treatment with the currently available, *in vivo* effective GABA_B_ PAMs (namely, CGP7930, GS39783, BHF177, *rac*-BHFF, and ADX71441). Specifically, repeated (once daily for 5–7 consecutive days) treatment with CGP7930 (25–100 mg/kg, i.g.), GS39783 (6.25–100 mg/kg, i.g.), and *rac*-BHFF (50–200 mg/kg, i.g.) has been reported to reduce daily alcohol intake in sP rats exposed to the 2-bottle “alcohol *vs* water” choice regimen (Orrù et al., 2005; Loi et al., 2013); daily water intake was compensatorily increased, leading to exclude that reduction in alcohol drinking was secondary to sedation. More recently, acute treatment with ADX71441 (3–30 mg/kg, i.g.) reduced alcohol intake in C57BL/6J mice exposed to (a) a model of excessive alcohol drinking based on the intermittent access, once every other day, to 20% (v/v) alcohol [this procedure is known to powerfully generate daily intakes of alcohol larger than 20 g/kg in C57BL/6J mice (Hwa et al., 2011)], and (b) the DID model of binge-like drinking (Hwa et al., 2014). Complete suppression of binge-like drinking generated in C57BL/6J mice by the DID model was also observed after acute treatment with 30 mg/kg GS39783 (i.p.) (Linsenbardt and Boehm, 2013).

#### Operant self-administration of alcohol

Several, recent studies have investigated the effect of GABA_B_ PAMs on operant, oral alcohol self-administration in alcohol-preferring rats. An initial study found that acute treatment with CGP7930 (10 and 20 mg/kg, i.p.) reduced lever-responding for alcohol in P rats exposed to an FR3 schedule of reinforcement (Liang et al., 2006). Subsequent studies, conducted with sP rats exposed to an FR4 schedule of reinforcement, reported the capacity of GS39783 (25–100 mg/kg, i.g.) (Maccioni et al., 2007), BHF177 (12.5–50 mg/kg, i.g.) (Maccioni et al., 2009), and *rac*-BHFF (50–200 mg/kg, i.g.) (Maccioni et al., 2010)—given under a Latin square design—to reduce lever-responding for alcohol. In the few studies in which baclofen and GABA_B_ PAMs were concurrently tested (Liang et al., 2006; Maccioni et al., 2012), baclofen resulted to be more potent and effective in reducing lever-responding for alcohol; however, at variance with the results collected with baclofen, the reducing effect of GS39783, BHF177, and *rac*-BHFF (at least within a given dose-range for the latter compound) displayed the important feature of being highly selective for alcohol self-administration: indeed, treatment with these compounds did not alter lever-responding for a sucrose solution in independent sets of sP rats. Additional studies are needed to understand the bases of the differential selectivity of the “*anti*-alcohol” effects of baclofen and GABA_B_ PAMs; nevertheless, these “selectivity” data are in close agreement with the results of several studies (Cryan et al., 2004; Malherbe et al., 2008; Paterson et al., 2008) suggesting the capacity of GABA_B_ PAMs to produce the “desired” behavioral effects—anxiolysis or “*anti*-addictive” effects—at doses far lower than those inducing motor-impairment and sedation, displaying higher therapeutic index and better side-effect profile than baclofen.

When tested in experiments of PR schedule of reinforcement, GS39783 (25-100 mg/kg, i.g.) (Maccioni et al., 2008b) and BHF177 (12.5–50 mg/kg, i.g.) (Maccioni et al., 2009)—given under a Latin square design—reduced lever-responding and breakpoint for alcohol in sP rats. Again, the reducing effect of both compounds was fully selective for alcohol, as no dose altered—even minimally—breakpoint for a sucrose solution.

A more recent study (Maccioni et al., 2012) compared the effect of GS39783 on alcohol self-administration in P, sP, and AA rats exposed to identical FR4 and PR schedules of reinforcement for alcohol: although treatment with GS39783 reduced number of lever-responses, amount of self-administered alcohol, and breakpoint in all rat lines, potency and efficacy of GS varied largely among the three lines, being highest in those rats (P line) in which alcohol displayed the strongest reinforcing and motivational properties and lowest in those rats (AA line) in which alcohol was less reinforcing and motivating. If ideally translated to humans, these data would suggest that GS39783 is more effective in those patients affected by severe forms of AUD.

A recent, confirmatory study reported the capacity of acutely administered BHF177 (3.75–30 mg/kg, i.p.) to reduce self-administration of a beer-like mixture, containing 9% alcohol, in C57BL/6J mice trained to lever-press under an FR3 schedule of responding (Orrù et al., 2012).

## Clinical data

### Baclofen and alcohol withdrawal syndrome

Clinical management of AWS is aimed at (a) reducing the severity of symptoms, (b) preventing more severe manifestations, such as seizures and *delirium tremens*, and (c) facilitating patient's admission into treatment programs to maintain long-term abstinence (Hall and Zador, 1997; Mayo-Smith, 1997). Clinical evaluation of AWS symptoms can be performed by means of several scales, including the Clinical Institute Withdrawal Assessment for Alcohol-revised scale (CIWA-Ar) (Mayo-Smith, 1997). This scoring system allows a quantitative evaluation of AWS severity and enhances identification of subjects potentially requiring pharmacological treatment. In addition to administration of fluids, thiamine, and electrolytes, benzodiazepines represent the current drugs of choice in the treatment of AWS (Amato et al., 2010, 2011). However, benzodiazepines may feature addictive properties, representing a major limitation to their use in subjects affected by AUD (Leggio et al., 2008).

Preliminary data based on case reports (Addolorato et al., 2002a, 2003), retrospective chart review (Stallings and Schrader, 2007), and randomized-controlled trials (Addolorato et al., 2006; Lyon et al., 2011) support the effectiveness of baclofen in the treatment of AWS in humans.

#### Case reports

A first study showed that baclofen administration rapidly suppressed symptoms of severe AWS (Addolorato et al., 2002a). In this study, 5 patients (4 men and 1 woman; mean daily drinks: 24; mean CIWA-Ar score: 27) were treated with relatively low oral doses of baclofen, equal to 30 mg/day [10 mg, administered three times a day (t.i.d.)], and AWS severity was evaluated every hour for 4-8 hours. After baclofen administration, all 5 patients showed a rapid decrease in CIWA-Ar scores and a rapid improvement of well-being. Patients were maintained at 30 mg/day baclofen, during a 30-day follow-up period, and all maintained abstinence without reporting significant side effects.

Addolorato et al. (2003) also reported the ability of 75 mg/day baclofen (25 mg t.i.d.) to stop *delirium tremens*, the most severe complication of AWS. Namely, a single baclofen administration decreased CIWA-Ar score below 8 within 1 h in a patient with severe AWS, complicated by *delirium tremens*. Effectiveness was confirmed by a progressive normalization, within 4 days of treatment, of biological markers of excessive alcohol consumption (e.g.: GGT, AST, ALT, and MCV). Following stabilization, baclofen dose was reduced to 30 mg/day, and the patient maintained full abstinence from alcohol over the subsequent 30 days, with no relevant side effects.

#### Comparative studies

In a subsequent study, Addolorato et al. (2006) compared the effectiveness of baclofen with benzodiazepines. Outpatients (18 subjects) were randomized to baclofen (30 mg/day for 10 consecutive days) or diazepam (19 subjects; 0.5–0.75 mg/kg/day for 6 consecutive days, and tapering of dose by 25% daily from day 7 to day 10). Both baclofen and diazepam significantly decreased CIWA-Ar scores, with no differences between the two treatments. However, evidence for recommending baclofen in the treatment of AWS was considered insufficient by a recent Cochrane review (Liu and Wang, 2011).

#### Retrospective studies

In a study carried out on data recorded at the Inpatient Medical Centre, St. Anthony's Hospital, Oklahoma City, Stallings and Schrader (2007) found that administration of baclofen prevented the development of symptoms of AWS in 12 cases out of 17. However, no detail was provided as to the dosage used.

#### Randomized, placebo-controlled studies

Finally, the results of a randomized, double-blind, placebo-controlled trial also confirmed the effectiveness of baclofen in reducing the intensity of AWS (Lyon et al., 2011). In this study, 44 subjects with symptoms of AWS were randomized to baclofen (30 mg/day) or placebo, and all subjects received symptom-triggered benzodiazepine treatments. Administration of baclofen was associated with a significant reduction in the use of benzodiazepines in the management of AWS over 72 h of observation.

### Baclofen to achieve and maintain alcohol abstinence

Despite the promising results on AWS treatment, studies conducted to date to evaluate its effectiveness in achieving and maintain abstinence have not achieved convergent results.

#### Case reports

A series of case reports found that the administration of doses of baclofen ranging from 75 to 400 mg/day (divided into 3 daily administrations) markedly reduced or completely suppressed alcohol intake in patients affected by AUD (Ameisen, 2005; Agabio et al., 2007; Bucknam, 2007; Dore et al., 2011). Interestingly, one of these articles described the personal history of a French physician, who conducted an original dose-finding curve with baclofen, with the aim of treating his own AUD (Ameisen, 2005). After having used all the available AUD medications (disulfiram, naltrexone, acamprosate, and topiramate), Dr. Olivier Ameisen tested oral baclofen, starting with the “conventional” dose of 30 mg/day. He then increased the daily dose of baclofen up to 270 mg (9 times higher than the “conventional” dose), experiencing—“*for the first time in my alcoholic life*”—complete abstinence and suppression of craving for alcohol. Because of somnolence, Ameisen progressively reduced the baclofen dose to 120 mg/day, adding 40 mg in stressful situations. This “maintenance” dose provided a complete control of his alcohol craving without any particular side effect. Ameisen also suffered from comorbid anxiety disorders, and the 120 mg/day baclofen “maintenance” dose completely suppressed anxiety. Other studies found that baclofen administration ameliorated anxiety in alcohol-dependent subjects suggesting that the “*anti*-alcohol” effects of baclofen in patients affected by comorbid anxiety disorders may be due, at least in part, to a relief of anxiety symptoms (Krupitsky et al., 1993; Addolorato et al., 2002b; Flannery et al., 2004; Garbutt et al., 2010).

Two of these case reports indicated that administration of high doses of baclofen was effective also in patients affected by AUD and other psychiatric illnesses, resistant to previous treatments (Agabio et al., 2007; Dore et al., 2011). Notably, these patients did not develop any side effects due to the combination of baclofen and other psychoactive drugs (namely, paroxetine, haloperidol, and benzodiazepines).

#### Observational studies

Two recent observational studies confirmed the effectiveness of high doses of baclofen (up to 330 mg/day; average effective dose: 147 mg/day) in reducing alcohol consumption (de Beaurepaire, 2012; Rigal et al., 2012). Namely, at 3-month observation, one study found that more than 80 out of 100 patients affected by AUD and resistant to usual treatment reduced alcohol consumption from the initial “high risk” to “low risk” or “moderate risk” level; at 2-year observation, 60% of these patients were still at “low risk” or “moderate risk” level (de Beaurepaire, 2012). The second study found that, after 1 year of treatment with high doses of baclofen, more than 80% of 132 patients initially classified as “high risk drinkers” were abstinent or reduced alcohol consumption to “low risk” level (Rigal et al., 2012).

#### Open studies

Two open studies evaluated the effectiveness of a lower dose of baclofen, equal to 30 mg/day, in the treatment of AUD (Addolorato et al., 2000; Flannery et al., 2004). Namely, the first trial recruited 10 men with AUD, and lasted 4 consecutive weeks (Addolorato et al., 2000); the second study recruited 12 subjects with AUD (3 women and 9 men), and lasted 12 consecutive weeks (Flannery et al., 2004). Both studies found that this dose of baclofen effectively reduced daily drinking, episodes of heavy daily drinking, and severity of craving for alcohol and anxiety. However, in the second study, no patient achieved and maintained abstinence (Flannery et al., 2004).

#### Randomized, placebo-controlled studies

Two series of randomized clinical trials, conducted in Italy and in the U.S., generated diverging results, using 30 mg/day baclofen (Addolorato et al., 2000, 2002b, 2007, 2011; Garbutt et al., 2010). According to the findings of the Italian studies, the oral administration of this dose of baclofen reduced alcohol consumption and craving for alcohol (Addolorato et al., 2000, 2002b, 2007, 2011), while the American study yielded less promising results (Garbutt et al., 2010). The first randomized, placebo-controlled, double-blind trial was conducted in Italy, and recruited 39 patients with AUD: 20 patients received baclofen (30 mg/day), and 19 patients received placebo (Addolorato et al., 2002b). Treatment lasted 4 consecutive weeks, and patients received psychological counseling once a week. The results of the study showed that baclofen treatment increased the number of patients displaying complete abstinence (14/20 and 4/19 in baclofen and placebo groups, respectively) and decreased alcohol consumption in patients who did not achieve complete abstinence [the number of daily drinks dropped from 18 (start of treatment) to less than 0.5 (end of treatment) in the baclofen group; it changed from 10 (start of treatment) to approximately 4 (end of treatment) in the placebo group]. Baclofen was also effective in reducing craving and anxiety scores.

The second randomized, placebo-controlled, double-blind trial was conducted in the U.S. (Garbutt et al., 2010). This study recruited 80 patients with AUD: 40 patients (22 and 18 men and women, respectively) received baclofen (30 mg/day), and 40 patients (22 and 18 men and women, respectively) received placebo. Treatment lasted 12 consecutive weeks, and patients received psychological support 8 times during the 12-week treatment. The results of this study showed that baclofen was not effective in reducing alcohol consumption, achieving abstinence, or reducing craving for alcohol. Conversely, baclofen treatment was associated with a reduction in anxiety scores.

These divergent results have been attributed, at least in part, to methodological differences between the two studies (e.g.: study duration, type and frequency of psychological support, number of women recruited) (Leggio et al., 2010). For instance, mean daily drinks at the start of baclofen treatment was equal to approximately 14 and 7 in the Italian and U.S. studies, respectively. This discrepancy indicates that the Italian study recruited patients with more severe AUD than the study conducted in the U.S. These differences suggest that baclofen may be more potent and effective in the treatment of severe AUD, rather than moderate AUD (Leggio et al., 2010).

This hypothesis could also explain the extremely high efficacy of relatively low doses of baclofen found in subjects affected by liver cirrhosis and AUD, in whom the severity of AUD was so severe as to impede them from achieving abstinence despite the liver disease (Addolorato et al., 2007). This randomized, placebo-controlled, double-blind trial recruited 84 patients (61 men and 23 women): 42 patients received baclofen (30 mg/day), and 42 patients received placebo, for 12 consecutive weeks. Patients received psychological support counseling every week for the first month, and then every 2 weeks. The results of this study showed that baclofen treatment increased the number of patients achieving and maintaining complete abstinence (30/42 and 12/42 in baclofen and placebo groups, respectively), the cumulative abstinence duration (defined as the number of cumulative days of total abstinence: 63 and 31 days in baclofen and placebo groups, respectively), and reduced alcohol-craving score.

Finally, a more recent randomized, placebo-controlled, double-blind trial did not achieve the preplanned level of participation (Addolorato et al., 2011). It was an international, multi-center study aimed at evaluating the effectiveness of two different doses of baclofen (30 and 60 mg/day). Unfortunately, due to methodological limitations, only the dataset collected in Italy could be analyzed; it was found that baclofen induced a dose-effect reduction of alcohol intake.

All together, the results obtained by randomized studies are less promising than those obtained by case reports. Among the several reasons that may have contributed to this discrepancy, the difference in the dose of baclofen used seems to be one of the most relevant. Namely, the majority of randomized trials used ¼ of the “maintenance” dose found to be concurrently effective and safe by Ameisen in his dose-finding curve. It is possible that the dose used by randomized trials was insufficient at least for certain patients. Additional studies should be conducted to investigate whether higher doses of baclofen may result in more satisfactory results in the treatment of AUD, taking into account the typology of patients (e.g., severe or moderate AUD), their gender, possible comorbid mental disorders, and/or renal function (see below).

To summarize, the limited number of studies conducted and the contrasting results obtained by these studies prevent the drawing of any conclusions regarding baclofen effectiveness in the treatment of AUD. However, nowadays in France an overwhelming number of patients affected by AUD ask for and receive high doses of baclofen to treat their disorder (Gorsane et al., 2012; Rolland et al., 2012, 2013; Pastor et al., 2013).

### Baclofen and other substances of abuse

Preclinical studies demonstrated that baclofen is effective in reducing the severity of opioid withdrawal syndrome in rats and mice; a single double-blind clinical trial, including 62 patients affected by opioid withdrawal syndrome, confirmed these results in humans (see Agabio et al., 2013). Conversely, despite a large body of preclinical studies suggesting the capacity of baclofen to reduce intravenous self-administration of cocaine, heroin, nicotine, and methamphetamine in rodents, clinical studies failed to extend these findings to humans (see Vlachou and Markou, 2010). Only one randomized, placebo-controlled study found that baclofen administration (80 mg/day) reduced the daily number of cigarettes in 60 patients affected by nicotine use disorder; conversely, four randomized, placebo-controlled studies found that the administration of baclofen (60 mg/day) failed to reduce the use of cocaine, opioids, and methamphetamine in 160, 40, and 88 patients, respectively, affected by substance use disorder (see Agabio et al., 2013).

Once again, the different results obtained by preclinical and clinical studies suggest that further studies are needed to investigate whether higher doses of baclofen may result in more satisfactory results in the treatment of substance use disorder from drugs other than alcohol.

## Safety profile of baclofen

### Pharmacokinetics

Baclofen is available in formulations intended for oral or intrathecal administration. The latter route of administration is used in the treatment of spasticity unresponsive to oral administration of baclofen (Dario and Tomei, 2004). The recommended oral daily dose for baclofen ranges from 15 to 80 mg, starting from 15 mg/day (5 mg, t.i.d.), and gradually increasing 5 mg t.i.d., every 3 days. Higher daily doses (up to 120 mg/day) should only be administered to hospitalized patients. After administration, baclofen is rapidly absorbed and up to 80% of an oral dose is excreted in the urine (related to creatinine clearance), with only a limited hepatic metabolism (Gerkin et al., 1986; Wuis et al., 1989). These pharmacokinetic characteristics make baclofen the ideal pharmacological treatment for alcoholic patients affected by liver disease while it should be avoided in patients with impaired renal function (Chen et al., 1997; Smith et al., 1999). Accordingly, renal function should be carefully assessed prior to baclofen administration (Agabio et al., 2013).

When administered in low or moderate doses, baclofen has a short half-life (2–6 h) and needs 3–4 daily administrations to maintain therapeutic effects (Lal et al., 2009). When administered in higher doses or in patients with impaired renal function, its half-life is longer, and lower daily administrations may be required (Gerkin et al., 1986; Chen et al., 1997; Smith et al., 1999).

### Side effects

The wide use of baclofen in the treatment of spasticity has underpinned the collection of information on its safety and side effects (Dario and Tomei, 2004). Generally, side effects observed after the administration of low or moderate doses of baclofen are mild and transient and include sedation or somnolence, excessive weakness, and vertigo (Agabio et al., 2013). Symptoms are rarely severe and frequently subside or disappear with continued therapy.

Low doses of baclofen have also proved to be manageable and safe to use in patients affected by AUD (Krupitsky et al., 1993; Addolorato et al., 2000, 2002a,b, 2003, 2007, 2011; Flannery et al., 2004; Garbutt et al., 2010). In these studies side effects were tolerable, mild to moderate—also in patients affected by liver cirrhosis—and no study reported the occurrence of a withdrawal syndrome after baclofen suspension or craving for baclofen. As sedation is the most common side effect associated to baclofen treatment, the additive sedative effects of baclofen and alcohol may theoretically constitute a problem for patients who do not achieve and maintain abstinence. However, no increase in sedation has been observed in baclofen-treated patients who continued to drink (Flannery et al., 2004; Bucknam, 2007; Garbutt et al., 2010; Rigal et al., 2012). The results of studies from two laboratories confirmed this empirical observation (Evans and Bisaga, 2009; Leggio et al., 2013). Taken together, these results suggest that the administration of low doses of baclofen may be considered safe even if subjects continue to drink.

Higher doses of baclofen are usually well tolerated if dosage is increased as well as discontinued gradually. Abrupt interruption and withdrawal from baclofen treatment may lead to possible onset of seizures, psychological symptoms, and hyperthermia (Dario and Tomei, 2004). For instance, a case of withdrawal *delirium* after baclofen discontinuation has been described in a patient affected by AUD who had received 75 mg/day baclofen for 6 months (Nasti and Brakoulias, 2011). The risk of withdrawal symptoms should be minimized by avoiding the abrupt discontinuation of therapy, reducing the dose by 5 mg, t.i.d., every 3 days, until termination of therapy under strict medical supervision (Agabio et al., 2013).

Intoxication after oral administration of baclofen is due to error of administration, attempted suicide or impaired renal function, and symptoms may be constituted by nausea, hypotonia, dizziness, respiratory depression, coma, seizures, cardiac conduction abnormalities, and EEG abnormalities (Lee et al., 1992; Dario and Tomei, 2004). Usually, the outcome of baclofen intoxication is good and a complete recovery of patients has been reported even after episodes of coma caused by the intrathecal administration of very high doses of baclofen (Agabio et al., 2013). Very few cases of voluntary abuse of baclofen have been described, execpt for attempting suicide (Lee et al., 1992; Perry et al., 1998; Weisshaar et al., 2012). To date, baclofen has not displayed addictive properties in patients affected by AUD.

## Conclusions

Several lines of experimental evidence consistently suggest that treatment with the prototypic GABA_B_ receptor agonist, baclofen, is effective in suppressing multiple alcohol-related behaviors—including excessive alcohol drinking, binge-like drinking, operant alcohol self-administration, reinstatement of alcohol-seeking behavior, and conditioned place preference—in rats, mice, and baboons exposed to experimental procedures modeling different aspects of human AUD. Treatment with baclofen also suppressed several signs of AWS in rats. Although still rather sporadic, the clinical data currently available suggest that the majority of these animal findings may be successfully extended to human alcoholics. The majority of these surveys suggest indeed that treatment with baclofen may promote abstinence and decrease alcohol consumption, craving for alcohol, and severity of alcohol withdrawal symptoms and signs, including *delirium tremens*, in alcohol-dependent patients. Additional studies are now needed to provide more definitive conclusions on the therapeutic potential of baclofen, including the high-dose treatment option inspired by Ameisen's work. Interestingly, the capacity of baclofen to be effective on both ameliorating AWS and achieving abstinence from alcohol is seen as a *plus*, since it may facilitate patients' admission into a long-term program aimed at maintaining abstinence without requiring a change of the pharmacological agent.

Finally, a major step forward in the pharmacology of the GABA_B_ receptor seems to be represented by GABA_B_ PAMs. Preclinical data collected to date, demonstrating the ability of GABA_B_ PAMs to reproduce the suppressive effects of baclofen on several alcohol-related behaviors, together with their more favorable safety profile, advocate the remarkable potential of this new class of agents. Notably, some GABA_B_ PAMs are currently approaching clinical evaluation (Addex Therapeutics webpage), leading to hypothesize that they might be tested, in a reasonably short period of time, in human alcoholics.

### Conflict of interest statement

The authors declare that the research was conducted in the absence of any commercial or financial relationships that could be construed as a potential conflict of interest. Giancarlo Colombo is one of the inventors of patent n. EP1217999 entitled “The use of baclofen in the treatment of alcoholism.”
